# Network Pharmacology and Molecular Docking Analyses of Mechanisms Underlying Effects of the *Cyperi Rhizoma*-*Chuanxiong Rhizoma* Herb Pair on Depression

**DOI:** 10.1155/2021/5704578

**Published:** 2021-12-22

**Authors:** Yanan Shi, Mingqi Chen, Zehua Zhao, Juhua Pan, Shijing Huang

**Affiliations:** ^1^Research and Development Center of Traditional Chinese Medicine, Guang'anmen Hospital, China Academy of Chinese Medical Sciences, Beijing 100053, China; ^2^Graduate School, Beijing University of Chinese Medicine, Beijing 100029, China

## Abstract

**Objective:**

We aimed to investigate the mechanisms underlying the effects of the *Cyperi Rhizoma*-*Chuanxiong Rhizoma* herb pair (CCHP) against depression using a network pharmacology approach.

**Methods:**

A network pharmacology approach, including screening of active compounds, target prediction, construction of a protein-protein interaction (PPI) network, gene ontology (GO) and Kyoto Encyclopedia of Genes and Genomes (KEGG) pathway enrichment analyses, and molecular docking, molecular dynamics (MD) simulations, and molecular mechanics Poisson–Boltzmann surface area (MMPBSA), were used to explore the mechanisms of CCHP against depression.

**Results:**

Twenty-six active compounds and 315 and 207 targets of CCHP and depression, respectively, were identified. The PPI network suggested that AKT1, IL-6, TP53, DRD2, MAPK1, NR3C1, TNF, etc., were core targets. GO enrichment analyses showed that positive regulation of transcription from RNA polymerase II promoter, plasma membrane, and protein binding were of great significance. Neuroactive ligand-receptor interaction, PI3K-Akt signaling pathway, dopaminergic synapse, and mTOR signaling pathway were important pathways. Molecular docking results revealed good binding affinities for the core compounds and core targets. MD simulations and MMPBSA validated that quercetin can stably bind to 6hhi.

**Conclusions:**

The effects of CCHP against depression involve multiple components, targets, and pathways, and these findings will promote further research on and clinical application of CCHP.

## 1. Introduction

Depression is a highly prevalent psychiatric illness with a global incidence of 258 million cases in 2017 and is predicted to be the leading contributor to worldwide disease burden by 2030, with a disability-adjusted life year (DALY) value of 84.32 million [1–3]. Depression has a major influence on individual health, is associated with high risks of lifetime suicide attempts, and imposes a heavy socioeconomic burden [1]. However, two-thirds of the patients prescribed antidepressant drugs do not show a beneficial treatment response [4].

Many studies have reported that traditional Chinese medicine (TCM) can treat depression effectively with fewer adverse events [6–8], since TCM treats depression from a holistic perspective with multiple ingredients and targets, rather than focusing on specific targets like other antidepressants [9]. Fewer adverse events of TCM also result from lower toxicities of herbs and further toxicity reduction by formulas, which are composed of interactive herbs [8].

Herb pairs used in TCM are composed of two herbs with synergistic effects; herb pairs are of particular clinical significance and provide an important perspective in studies of herb compatibility [10]. The combination of *Cyperi Rhizoma* and *Chuanxiong Rhizoma* is deemed the *Cyperi Rhizoma*-*Chuanxiong Rhizoma* herb pair (CCHP) in TCM [11]. It is an important part of the famous formulas widely used in treating depression, including the Yueju pill from *Danxi Xinfa* and Chaihu Shugan San from *Jingyue Quanshu*. Both Yueju pill and Chaihu Shugan San were found to exert significant antidepressant effects in clinical trials [12–15]. Yueju pill exerted antidepressant effects by regulating PKA/CREB, NMDA, and Akt/mTOR signaling [16–18], while Chaihu Shugan San has been suggested to treat depression through BDNF signaling, gut microbiota, and other mechanisms [19, 20]. However, the compatible mechanisms underlying the therapeutic effects of CCHP require further research.

Network pharmacology is a new approach derived from systems biology, polypharmacology, network theory, and so on. TCM is characterized by holism, and TCM formulas are known to treat diseases by employing multiple ingredients and targets from a systematic perspective. Since the properties of network pharmacology are in accordance with the holistic philosophy of TCM, network pharmacology offers a new approach to innovating drug discovery and an effective tool for exploring TCM from a systematic perspective [21, 22]. The combination of TCM and network pharmacology can elucidate the underlying mechanisms at the molecular level and systematically illustrate complicated biological network relationships [22, 23].

**Figure 1:**
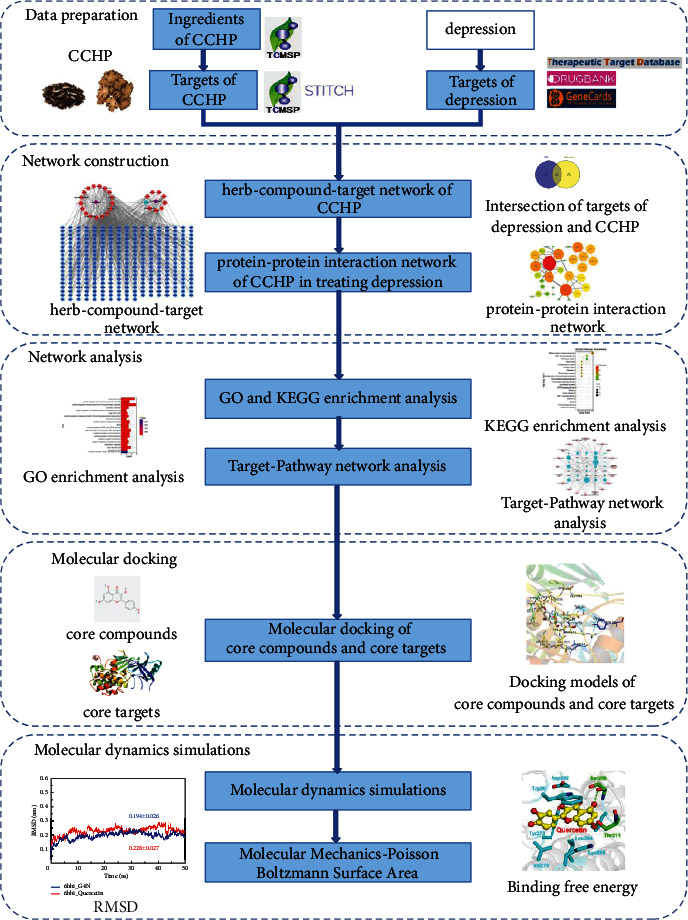
Workflow for the network pharmacology-based study of CCHP in treating depression.

Thus, the purpose of this study was to investigate the multiple mechanisms of CCHP in treating depression using network pharmacology and molecular docking to provide insights into the research and therapy of depression. A detailed workflow is shown in Figure 1.

## 2. Materials and Methods

### 2.1. Acquisition of the Active Compounds of CCHP

The active compounds of CCHP were predominantly retrieved from the Traditional Chinese Medicine Systems Pharmacology Database and Analysis Platform (TCMSP, https://tcmspw.com/tcmsp.php). The core compounds of CCHP that were recorded in the literature and not included in TCMSP were also obtained. TCMSP can provide information on the ingredients, corresponding targets, and pharmacokinetic properties of TCM [24]. The database provides pharmacokinetic information, such as drug-likeness (DL) and oral bioavailability (OB). The screening thresholds of compounds retrieved from TCMSP were set as OB ≥ 30% and DL ≥ 0.18 [25]. Compounds without target information were removed.

### 2.2. Prediction of the Targets of Active Compounds

We used TCMSP and the search tool for interacting chemicals (STITCH, http://stitch.embl.de/) to acquire the targets of each compound [25]. In STITCH, we selected “*Homo sapiens*” as the species and chose targets with a combined score of ≥0.7. The targets of the compounds obtained were standardized in the UniProt (https://www.uniprot.org) database, and “reviewed” and “human” UniProtKB was selected [26]. Then, the duplicated targets were removed from the targets obtained.

### 2.3. Construction of the Herb-Compound-Target Network

To illustrate the relationships between herbs, compounds, and targets of CCHP, Cytoscape 3.2.1 Software (http://www.cytoscape.org/) [27] was utilized to build a herb-compound-target network.

### 2.4. Acquisition of Targets Related to Depression

Targets related to depression were retrieved from the therapeutic target database (TTD, https://db.idrblab.org/ttd/) [28], DrugBank (https://www.drugbank.ca/) [29], and GeneCards Version 5.1 (https://www.genecards.org/) [30] databases with the keyword “Depression.” In GeneCards, targets with a score of ≥16 were screened.

### 2.5. Intersection of Targets of Depression and CCHP

To obtain the targets of CCHP in treating depression, the predicted targets of the compounds of CCHP were intersected with targets related to depression, and a Venn diagram was obtained using the Venny 2.1 (http://bioinfogp.cnb.csic.es/tools/venny/index.html) mapping tool.

### 2.6. Protein-Protein Interaction Network Construction and Core Target Screening

To illuminate the interactions among proteins, the targets of CCHP in treating depression were input into STRING 11.0 (https://string-db.org/) for protein-protein interaction (PPI) analysis [31]. The parameters were set as follows: “*Homo sapiens*” was chosen as the species, and a combined score >0.9 was used as the threshold. The results for the PNG and TSV formats were exported. The PPI network was visualized by Cytoscape 3.2.1 and analyzed using the “Network analyzer” plug-in, which is a tool of Cytoscape. The screening thresholds were the median values of the degrees of all nodes.

### 2.7. Gene Ontology and Kyoto Encyclopedia of Genes and Genomes Pathway Enrichment Analyses

The Database for Annotation, Visualization, and Integrated Discovery (DAVID) v6.8 (https://david.ncifcrf.gov/) [32, 33] was utilized for gene ontology (GO) and Kyoto Encyclopedia of Genes and Genomes (KEGG) pathway enrichment to illuminate the biological function and enriched pathways of targets of CCHP in treating depression, with a screening criterion of *p* < 0.01 and false discovery rate (FDR) <0.05.

### 2.8. Construction of the Target-Pathway Network

Based on KEGG analysis, Cytoscape was employed to construct a target-pathway network of the top 20 key signaling pathways and the enriched targets. The relationships between pathways and enriched targets are shown in the network. The network nodes are the pathways and enriched targets, and the size of the nodes represents the topological importance of the nodes.

### 2.9. Molecular Docking

The nodes with the top six degrees of the herb-compound-target network and PPI network were chosen as core compounds and targets for molecular docking. First, the 2D structures of the core compounds were acquired from the PubChem database (https://pubchem.ncbi.nlm.nih.gov/) [34] and input into the ChemBio 3D Software to export the 3D structures. AutoDockTools 1.5.6 Software was then employed to add charge values and export the structures in pdbqt format. Second, the 3D structures of the core targets were acquired from the RCSB PDB database (https://www.rcsb.org/) [35] and deleted water and other ligands. AutoDockTools 1.5.6 was used to add hydrogen and charges and convert the structures into pdbqt format. Finally, AutoDock Vina 1.1.2 was utilized to perform molecular docking and analyze the results [36]. Docking results were visualized and analyzed using PyMOL 1.7.2.1 and Ligplus 2.2.4. The docking of core compounds and targets with lower docking energies had stronger binding forces.

### 2.10. Molecular Dynamics Simulations

Since AKT1 (PDB ID: 6hhi) was the core target and quercetin was the core compound, the docking conformation of 6hhi and quercetin, which had low binding energy, was selected as the initial conformation for molecular dynamics (MD) simulations. G4N, the primitive ligand of 6hhi, was used as the positive control. MD simulations were performed using the GROMACS 2018.4 program [37] under constant temperature and pressure and periodic boundary conditions. Amber99 SB all-atom force field and TIP3P water model were applied [38]. During MD simulations, all bonds involving hydrogen atoms were constrained using the LINear Constraint Solver (LINCS) algorithm [39] with an integration step of 2 fs. Electrostatic interactions were calculated using the particle mesh Ewald (PME) method [40]. The nonbonded interaction cutoff was set to 10 Å and updated every 10 steps. The V-rescale temperature coupling method [41] was used to control the simulation temperature at 300 K, and the Parrinello–Rahman method [42] was used to control the pressure at 1 bar.

First, energy minimization was performed in the two systems using 5000 steps of steepest descent algorithm with the convergence of energy minimization of 100 kJ/mol/nm to eliminate excessive interatomic contact. Then, the systems were heated gradually from 0 to 300 K in the canonical ensemble (NVT) and equilibrated at 300 K for 1000 ps in the constant pressure-constant temperature ensemble (NPT). Finally, the systems were subjected to MD simulations for 50 ns and the conformation was preserved every 10 ps. The simulation results were visualized using the GROMACS embedding program and visual molecular dynamics (VMD).

### 2.11. Calculation of Binding Free Energy

The molecular mechanics Poisson–Boltzmann surface area (MMPBSA) method [43] was used to calculate the binding energy between substrate small molecules and proteins in the two protein systems.

## 3. Results

### 3.1. Acquisition of the Active Compounds and Targets of CCHP

A total of 26 compounds of CCHP were acquired from TCMSP and the literature.

Among the compounds, 18 were from *Cyperi Rhizoma* and 9 were from *Chuanxiong Rhizoma*. The details of the compounds in each herb are shown in Table 1. By searching TCMSP and STITCH, 315 targets of the CCHP compounds were acquired, which included 302 targets of *Cyperi Rhizoma* and 73 targets of *Chuanxiong Rhizoma*. *Cyperi Rhizoma* and *Chuanxiong Rhizoma* shared 59 targets that may mediate their synergistic effects.

### 3.2. Construction and Analysis of the Herb-Compound-Target Network

**Figure 2:**
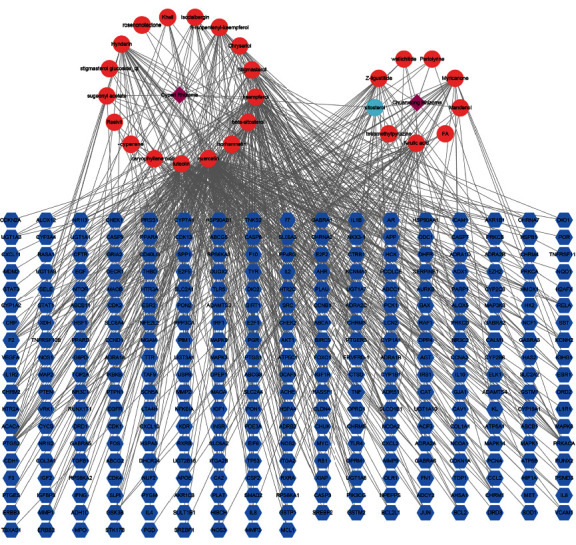
Herb-compound-target network of CCHP. Purple diamonds stand for the herbs; red ellipses represent the compounds of herbs; light blue ellipse stands for the common compounds of the two herbs; blue hexagons represent the targets of the compounds; and edges represent interactions between compounds and the corresponding targets or herbs.

The herb-compound-target network (Figure 2) built by Cytoscape contained 343 nodes and 762 edges. A Cytoscape network analyzer was used to perform topological analysis of the network. In the network, the degree represents the number of nodes that are directly connected to one node. Therefore, nodes with larger degrees may be key compounds or targets that play important roles in the network and were screened and further analyzed. As shown in the network, one compound may act on many targets, and numerous compounds may correspond to the same target. Considering the degrees of the compounds, MOL000098 (quercetin), MOL000006 (luteolin), MOL000422 (kaempferol), MOL000358 (beta-sitosterol), and MOL000354 (isorhamnetin) are pivotal compounds.

### 3.3. Intersection of the Targets of Depression and CCHP

We retrieved 207 targets related to depression from the TTD, DrugBank, and GeneCards databases (Additional File 1: Table S1). The targets of CCHP were intersected with targets related to depression to obtain the targets of CCHP in treating depression, and 40 overlapping targets were obtained using this approach (Table 2, Additional File 2: Figure S1).

### 3.4. PPI Network Construction and Core Target Analyses

**Figure 3:**
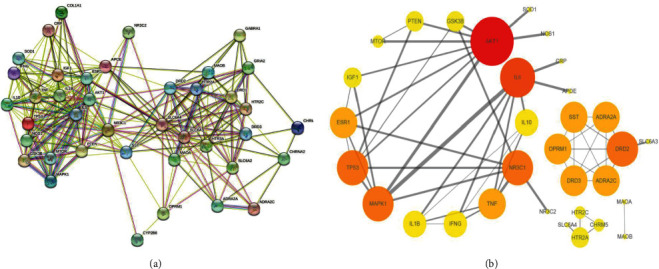
PPI network of CCHP in treating depression. (a) PPI network constructed by STRING. (b) PPI network constructed by Cytoscape. Nodes represent targets, and edges stand for the interactions between the targets. In Figure 3(b), with an increase in the degrees, the colors of the nodes change from yellow to red, and the sizes of the nodes increase.

The STRING database was utilized to analyze the interactions of these overlapping targets and construct the PPI diagram (Figure 3(a)) with an average node degree of 12.8 and a PPI enrichment *p* value of <1.0*e* − 16.

Targets with a combined score >0.9 were screened and input into Cytoscape to visualize and analyze the PPI network (Figure 3(b)). Topological analysis of the PPI network was performed using the Cytoscape Network Analyzer. The network included 32 nodes and 57 edges. The screening criteria for core targets were the median values of degree. The core targets obtained were AKT1, IL-6, TP53, DRD2, MAPK1, NR3C1, TNF, ESR1, SST, OPRM1, DRD3, ADRA2A, and ADRA2C.

### 3.5. GO Enrichment Analyses

**Figure 4:**
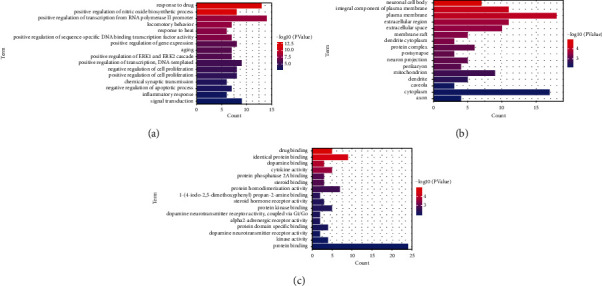
Top 16 GO enrichment analyses. The *x*-axis represents enrichment gene count, the *y*-axis represents terms related to BP, CC, or MF, and the color of the bar chart indicates the adjusted −log10 (*p* value). (a) BP. (b) CC. (c) MF.

GO enrichment analyses were performed by the DAVID. On the basis of the screening criteria of *p* < 0.01, 146 items were obtained, including 114 entries for biological process (BP), 16 entries for cellular component (CC), and 16 entries for molecular function (MF). The top 16 entries in BP analysis included positive regulation of transcription from RNA polymerase II promoter, response to drug, positive regulation of transcription (DNA-templated), and signal transduction (Figure 4(a)). The top 16 entries in CC analysis included the plasma membrane, cytoplasm, integral component of the plasma membrane, and the extracellular region (Figure 4(b)). In MF analysis, protein binding was the term that targets were predominantly enriched in Figure 4(c).

### 3.6. KEGG Pathway Enrichment Analyses

**Figure 5:**
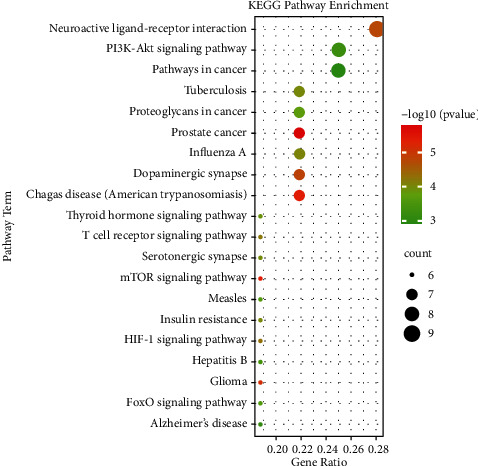
Top 20 KEGG pathway enrichment analyses. The *x*-axis represents enrichment gene ratio, and the *y*-axis represents terms related to KEGG pathway. The bubble size indicates enrichment gene count. The color stands for the adjusted *p* value.

KEGG pathway enrichment analyses were performed using the DAVID with the screening criterion of *p* < 0.01, and 51 pathways were obtained. The top 20 significantly enriched pathways included neuroactive ligand-receptor interaction (hsa04080), PI3K-Akt signaling pathway (hsa04151), pathways in cancer (hsa05200), dopaminergic synapse (hsa04728), and mTOR signaling pathway (hsa04150). The top 20 enriched pathways are displayed in detail in Figure 5.

### 3.7. Construction of the Target-Pathway Network

**Figure 6:**
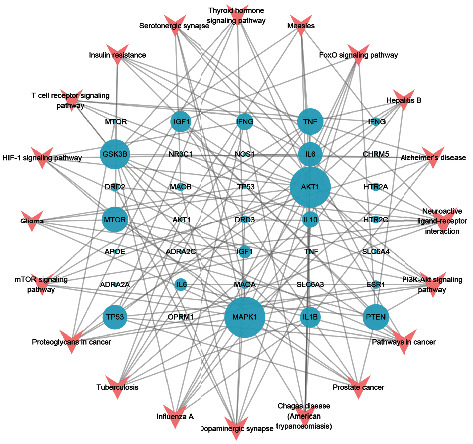
Target-pathway network of CCHP in treating depression. The pink V shapes represent the pathways. The light blue ellipses represent the core targets of CCHP in treating depression. The sizes of nodes indicate their degrees.

We input the top 20 key pathways and the enriched targets into Cytoscape to construct and analyze the target-pathway network (Figure 6). The degree was selected to assess the importance of the nodes. AKT1, MAPK1, GSK3B, TNF, MTOR, and PTEN had larger degrees and were core targets enriched in these pathways in the network. Neuroactive ligand-receptor interaction (hsa04080), pathways in cancer (hsa05200), and the PI3K-Akt signaling pathway (hsa04151) had larger degrees than other pathways.

### 3.8. Molecular Docking of Core Compounds and Core Targets

**Figure 7:**
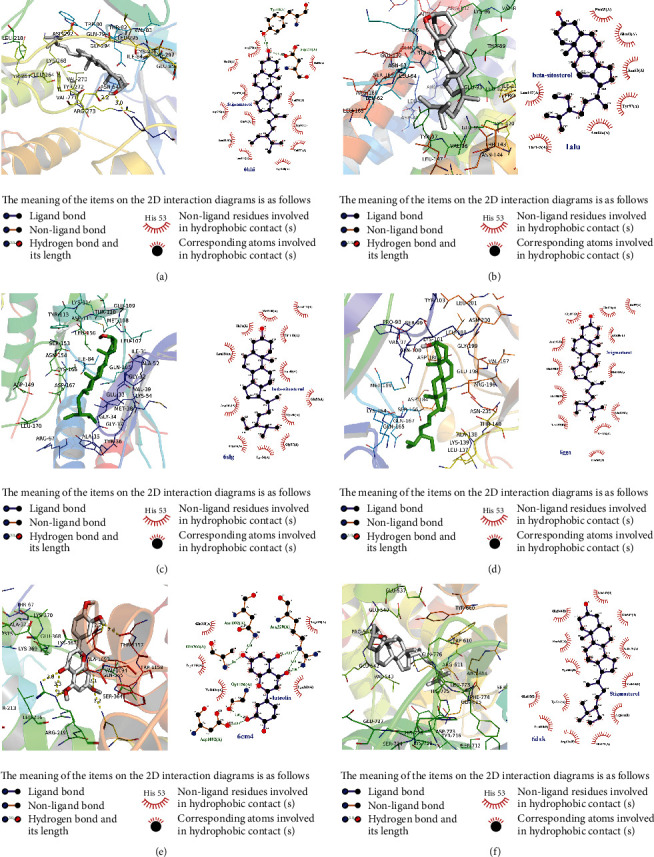
Docking models of core compounds and core targets. The left side of each picture displays the 3D interaction diagrams of the compounds and the targets. The compounds are represented by sticks. The targets are displayed in the ribbon model, yellow dashed lines represent the hydrogen bonds, and binding site residues are displayed in lines and labeled with amino acid codes. The right side of each picture shows the 2D interaction diagrams of the compounds and targets. The meaning of the items on the 2D interaction diagrams is shown in the legend. (a) AKT1 and stigmasterol. (b) IL-6 and beta-sitosterol. (c) MAPK1 and beta-sitosterol. (d) TP53 and stigmasterol. (e) DRD2 and luteolin. (f) NR3C1 and stigmasterol.

Molecular docking aims to predict the interactions between proteins and small molecules. The core compounds were quercetin, luteolin, kaempferol, beta-sitosterol, isorhamnetin, and stigmasterol. The core targets were AKT1 (PDB ID: 6hhi) [44], IL-6 (PDB ID: 1alu) [45], TP53 (PDB ID: 6gga) [46], DRD2 (PDB ID: 6cm4) [47], MAPK1 (PDB ID: 6slg) [48], and NR3C1 (PDB ID: 6dxk) [49]. As shown in Table 3, the binding energy values of the core compounds in CCHP with the core targets are less than −5 kcal/mol, indicating strong affinity. A lower binding energy indicates a stronger binding force. As shown in Figure 7, the core compounds are strongly bound to the core targets by forming hydrophobic and polar interactions.

### 3.9. MD Simulations

**Figure 8:**
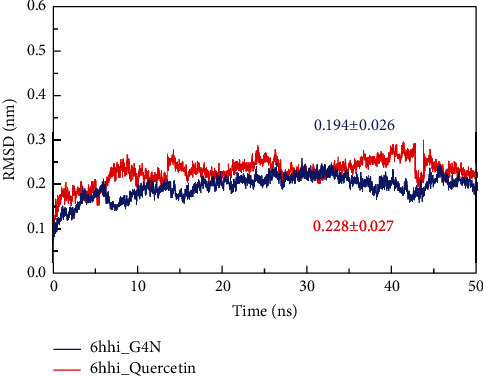
Root-mean-square deviation (RMSD) of 6hhi_Quercetin and 6hhi_G4N.

Root-mean-square deviation (RMSD) indicates the sum of all atomic deviations between the conformation at a certain time and the target conformation, which is an important basis for measuring the stability of the system. The system of the binding complex of 6hhi and its primitive ligand G4N was named 6hhi_G4N, and the system of the binding complex of 6hhi and quercetin was named 6hhi_Quercetin.  Figure 8 shows that the RMSD values of all C*α* atoms in the 6hhi_G4N and 6hhi_Quercetin systems change with time. The two systems basically tended to be stable after 10 ns, with the mean RMSD values of 0.194 ± 0.026 nm and 0.228 ± 0.027 nm, respectively. The RMSD fluctuations of both systems are small. In particular, the RMSD values of the 6hhi_Quercetin system are significantly higher than those of the 6hhi_G4N system from 5 ns, which may be due to the differences in small molecule compounds bound in the 6hhi protein that affect the stability of the entire complex to some extent.

**Figure 9:**
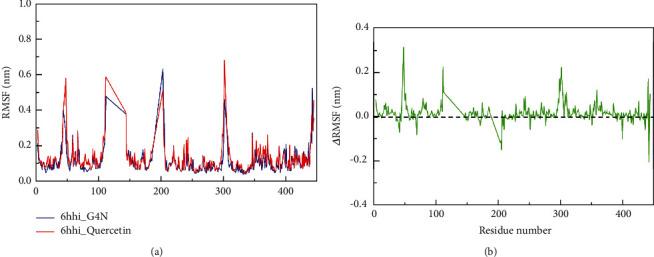
Root-mean-square fluctuations (RMSFs) per amino acid (aa) of 6hhi_Quercetin and 6hhi_G4N. (a) RMSF distribution of 6hhi_Quercetin and 6hhi_G4N. (b) RMSF change in 6hhi_Quercetin relative to 6hhi_G4N.

Root-mean-square fluctuations (RMSFs) can indicate the flexibility of amino acid residues in proteins. The amino acid flexibility distribution of 6hhi_G4N and 6hhi_Quercetin is shown in Figure 9. After the binding of quercetin, the flexibility of most amino acids of the 6hhi shows a significant increase (ΔRMSF > 0). The above results show that the RMSF of most amino acids of 6hhi increases slightly after the binding of quercetin compared with the previous 6hhi_G4N system. The increase in RMSF may be due to the differences in the key amino acids of the interactions between the two molecules.

### 3.10. Calculation of Binding Free Energy

The results of MMPBSA show that the binding energy of the substrate and protein in 6hhi_G4N (binding energy = −125.522 ± 14.620 kJ/mol) is higher than that in 6hhi_Quercetin (binding energy = −103.144 ± 10.692 kJ/mol) (Table 4). The results showed that both quercetin and G4N could stably bind to the active pocket of 6hhi, and G4N had stronger interactions with 6hhi than quercetin.

## 4. Discussion

Depression, as a highly prevalent psychiatric illness, has serious effects on physical and mental health and can even lead to suicide [50]. Although some antidepressants are effective, they often cause adverse effects and are expensive [5]. Chinese herbal medicine has been proven to be effective in treating depression through multiple components, targets, and pathways [8]. CCHP is the core component of many famous formulas that have significant curative effects on depression. We employed a network pharmacology approach to investigating the multiple mechanisms of CCHP in treating depression.

We obtained compounds and corresponding targets from the TCMSP and STITCH databases. Sitosterol was a common compound in *Cyperi Rhizoma* and *Chuanxiong Rhizoma*. Quercetin, a flavonoid, is present in numerous plants and exerts antidepressant effects by regulating the signaling related to BDNF [51, 52], alleviating oxidative stress and neuroinflammation [53], and inhibiting astrocyte reactivation [54]. Similarly, luteolin is a flavonoid with various biological properties [55]. The mechanisms underlying the antidepressant-like effect of luteolin may include the inhibition of endoplasmic reticulum stress [55, 56] and the regulation of monoaminergic and cholinergic functions [57].

The herb-compound-target network (Figure 2) showed that the relationships between the compounds and their corresponding targets were complicated. Quercetin, luteolin, kaempferol, beta-sitosterol, and isorhamnetin had larger degrees than other compounds, and they were core compounds in the network. One compound can act on several targets, and numerous compounds may share a common target. Therefore, we can infer that multiple compounds of CCHP may act on depression through multiple targets.

Targets of depression were retrieved from the TTD, DrugBank, and GeneCards databases; the targets of depression and CCHP were intersected; 40 targets were identified as targets of CCHP in treating depression (Additional File 2: Figure S1).

The targets of CCHP in the treatment of depression showed numerous interactions. The PPI network of CCHP in treating depression (Figure 3(b)), which was visualized and analyzed using Cytoscape, illustrated that AKT1, IL-6, TP53, DRD2, MAPK1, NR3C1, TNF, ESR1, SST, OPRM1, DRD3, ADRA2A, and ADRA2C were core targets of CCHP in treating depression. AKT1, an isoform of the serine/threonine kinase [58], is involved in monoamine neurotransmission and plasticity [59, 60]. A reduction in the kinase activity of AKT1 is related to depression [61]. *AKT1* gene polymorphisms may be a sign of depression severity and treatment response [58, 62, 63]. Crosstalk between inflammation and neurocircuits may be related to the pathogenesis and treatment response of depression [64]. IL-6 is a cytokine that is significantly associated with depression [65]. An increase in IL-6 levels has been observed in both the acute and chronic stages of depression [66]. IL-6 levels were significantly elevated via pivotal inflammatory signaling in response to stressors [67, 68]. TP53, which is called the mammalian cell “gatekeeper,” is a pro-apoptotic factor [69, 70] that plays a critical role in regulating astrocytic autophagy and neuronal apoptosis, which may explain the mechanisms underlying the antidepressant effects of fluoxetine [70, 71]. The dopaminergic system may be related to the pathogenesis of depression and the response to antidepressants [72]. DRD2 is a pivotal protein in the dopaminergic system [73]. The vulnerability to depression and reactivity of antidepressants are associated with *DRD2* gene polymorphisms [73–75]. MAPK1, which is involved in regulating neuroplasticity and inflammatory processes, appears to reflect vulnerability to depression [76, 77]. *MAPK1* polymorphisms may be related to the remission of antidepressant treatment [77].

The results of GO analysis are shown in Figure 4. BP analysis (Figure 4(a)) indicated that targets related to the regulation of transcription and gene expression, response to drug, signal transduction, positive regulation of nitric oxide biosynthetic process, and the regulation of cell proliferation were largely enriched. CC terms (Figure 4(b)) were mostly related to the plasma membrane, cytoplasm, extracellular region, and cytosol. MF terms (Figure 4(c)) were primarily related to protein binding.

As shown in Figure 5, neuroactive ligand-receptor interaction (hsa04080), PI3K-Akt signaling pathway (hsa04151), dopaminergic synapse (hsa04728), mTOR signaling pathway (hsa04150), and HIF-1 signaling pathway (hsa04066), which enriched many targets, may contribute to the antidepressant effects of CCHP. Neuroactive ligand-receptor interaction signaling contributes to the transmission of extracellular signals into cells [78]. This pathway, which includes numerous receptors and ligands, is linked to the mechanism of depression and the antidepressant effects of many TCM formulas [78–82]. PI3K/Akt signaling, which is activated by neuroinflammation, leads to neuroplastic damage in depression [83]. PI3K/Akt signaling may regulate neuroinflammatory factors and neurotrophins and exert antidepressant effects [84]. Inhibition of PI3K/Akt signaling plays a role in the neuroprotective effects of fluoxetine [85]. BDNF/TrkB activates PI3K/Akt signaling during antidepressant action [86]. The depletion of monoamine neurotransmitters is the pathophysiological basis of depression [87]. Decreased dopaminergic transmission may contribute to blunted reward processing and repaired reward learning, which are features of depression [88–90]. The antidepressant effects of dopamine agonists may depend on the ventrostriatal dopamine and reward function [91]. mTOR signaling, as a downstream intracellular signal, is associated with antidepressant effects [92, 93]. Fast-acting antidepressants, such as ketamine, enhance mTOR function and improve neurogenesis and plasticity [94, 95]. HIF-1 mediates mitochondrial metabolism, reduces oxidative stress, and plays a role in energy supply in depression [96–98]. Upregulation of HIF-1 may provide a new approach to antidepressant treatment [96].

The target-pathway network illustrated that AKT1, MAPK1, GSK3B, TNF, MTOR, and PTEN were core targets enriched in key signaling pathways that played crucial roles in the treatment of depression by CCHP. GSK3B may be involved in the development of depression by inhibiting Erk-CREB-BDNF signaling [99], and PI3K/Akt/mTOR/GSK3B signaling may be the mechanism underlying the rapid antidepressant effects [100]. TNF polymorphisms are associated with depression [65], and the suppression of TNF-*α*/TNFR1/NF-*κ*B signaling alleviated neuroinflammation and depression [101].

Molecular docking was employed to validate the interactions between the core compounds of CCHP and the core targets, and affinity analyses were used to estimate the binding energy of a ligand and the intensity of the interactions. The results indicated that multiple core compounds of CCHP could bind to multiple core targets, and this may be the basis of the mechanism underlying the therapeutic effects of CCHP.

MD simulations are able to predict the motion of each atom over time and refine the conformation of the receptor-ligand complex [102–104]. MD simulation in combination with binding free energy calculation can make the binding free energy estimates precise and re-rank the candidates [105]. MD simulation and MMPBSA results showed that quercetin can stably bind to the active pocket of 6hhi.

Nevertheless, this study had some limitations. The compound and target information used in the evaluations was mainly obtained from databases; however, some bioactive ingredients and targets may not be included in the databases. The inhibitory and activated effects of the targets are difficult to differentiate. The ingredients obtained from the databases may be distinct from those absorbed and utilized in the patient's body. Moreover, potential complex interactions between the ingredients were not taken into consideration. Accordingly, further experimental verification of the multiple mechanisms of CCHP in treating depression both in vivo and in vitro is required to validate the obtained results.

## 5. Conclusions

In this study, quercetin, luteolin, kaempferol, beta-sitosterol, and isorhamnetin were the core compounds of CCHP in treating depression. CCHP treated depression by acting on multiple key targets, such as AKT1, IL-6, TP53, DRD2, MAPK1, NR3C1, TNF, ESR1, SST, OPRM1, DRD3, ADRA2A, and ADRA2C, and through important biological processes, such as positive regulation of transcription from RNA polymerase II promoter, plasma membrane, and protein binding. CCHP exerted antidepressant effects by regulating multiple key signaling pathways such as neuroactive ligand-receptor interaction, PI3K-Akt signaling pathway, dopaminergic synapse, and mTOR signaling pathway. AKT1, MAPK1, GSK3B, TNF, MTOR, and PTEN were the core targets enriched in key signaling pathways. CCHP treats depression through multiple components, targets, and pathways. However, the specific antidepressant effects of CCHP require experimental verification.

## Figures and Tables

**Table 1:** Active compounds of CCHP.

MOL ID	Compound name	Number of targets	Herb
MOL000098	Quercetin	177	*Cyperi Rhizoma*
MOL000006	Luteolin	95	*Cyperi Rhizoma*
MOL000422	Kaempferol	93	*Cyperi Rhizoma*
MOL000354	Isorhamnetin	46	*Cyperi Rhizoma*
MOL000358	Beta-sitosterol	46	*Cyperi Rhizoma*
MOL000449	Stigmasterol	38	*Cyperi Rhizoma*
MOL004071	Hyndarin	33	*Cyperi Rhizoma*
MOL000360	Ferulic acid	32	*Chuanxiong Rhizoma*
MOL003542	8-Isopentenyl-kaempferol	28	*Cyperi Rhizoma*
MOL002135	Myricanone	25	*Chuanxiong Rhizoma*
MOL002122	Z-Ligustilide	23	*Chuanxiong Rhizoma*
MOL003044	Chrysoeriol	19	*Cyperi Rhizoma*
MOL000359	Sitosterol	13	*Cyperi Rhizoma, Chuanxiong Rhizoma*
MOL004053	Isodalbergin	12	*Cyperi Rhizoma*
MOL004344	Caryophyllene oxide	11	*Cyperi Rhizoma*
MOL004058	Khell	7	*Cyperi Rhizoma*
MOL004077	Sugeonyl acetate	7	*Cyperi Rhizoma*
MOL002202	Tetramethylpyrazine	6	*Chuanxiong Rhizoma*
MOL010489	Resivit	4	*Cyperi Rhizoma*
MOL002140	Perlolyrine	4	*Chuanxiong Rhizoma*
MOL002157	Wallichilide	4	*Chuanxiong Rhizoma*
MOL007508	*α*-Cyperene	3	*Cyperi Rhizoma*
MOL000433	FA	3	*Chuanxiong Rhizoma*
MOL001494	Mandenol	3	*Chuanxiong Rhizoma*
MOL004074	Stigmasterol glucoside_qt	2	*Cyperi Rhizoma*
MOL004068	Rosenonolactone	1	*Cyperi Rhizoma*

**Table 2:** Targets of CCHP in treating depression.

Gene symbol	Protein name	UniProt ID
AKT1	RAC-alpha serine/threonine-protein kinase	P31749
IL-6	Interleukin-6	P05231
TP53	Cellular tumor antigen p53	P04637
DRD2	D(2) dopamine receptor	P14416
MAPK1	Mitogen-activated protein kinase 1	P28482
NR3C1	Glucocorticoid receptor	P04150
TNF	Tumor necrosis factor	P01375
ESR1	Estrogen receptor	P03372
SST	Somatostatin	P61278
OPRM1	Mu-type opioid receptor	P35372
DRD3	D(3) dopamine receptor	P35462
ADRA2A	Alpha-2A adrenergic receptor	P08913
ADRA2C	Alpha-2C adrenergic receptor	P18825
IL-10	Interleukin-10	P22301
IL-1B	Interleukin-1 beta	P01584
IFN-G	Interferon-gamma	P01579
GSK3B	Glycogen synthase kinase-3 beta	P49841
PTEN	Phosphatidylinositol 3,4,5-trisphosphate 3-phosphatase and dual-specificity protein phosphatase PTEN	P60484
IGF1	Insulin-like growth factor I	P05019
HTR2A	5-hydroxytryptamine receptor 2A	P28223
MTOR	Serine/threonine-protein kinase mTOR	P42345
CHRM5	Muscarinic acetylcholine receptor M5	P08912
HTR2C	5-hydroxytryptamine receptor 2C	P28335
SLC6A3	Sodium-dependent dopamine transporter	Q01959
CRP	C-reactive protein	P02741
APOE	Apolipoprotein E	P02649
SOD1	Superoxide dismutase [Cu-Zn]	P00441
MAOA	Amine oxidase [flavin-containing] A	P21397
MAOB	Amine oxidase [flavin-containing] B	P27338
NOS1	Nitric oxide synthase, brain	P29475
NR3C2	Mineralocorticoid receptor	P08235
SLC6A4	Sodium-dependent serotonin transporter	P31645
CHRNA2	Neuronal acetylcholine receptor subunit alpha-2	Q15822
COL1A1	Collagen alpha-1(I) chain	P02452
CYP2B6	Cytochrome P450 2B6	P20813
DRD1	D(1A) dopamine receptor	P21728
GABRA1	Gamma-aminobutyric acid receptor subunit alpha-1	P14867
GRIA2	Glutamate receptor 2	P42262
HTR3A	5-hydroxytryptamine receptor 3A	P46098
SLC6A2	Sodium-dependent noradrenaline transporter	P23975

**Table 3:** Docking results of core compounds and core targets.

Proteins	PDB ID	Protein structure	Compounds	Affinity (kcal/mol)
AKT1	6hhi	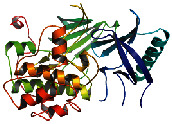	Quercetin	−9.4
			Luteolin	−9.7
			Kaempferol	−9.3
			Beta-sitosterol	−10.9
			Isorhamnetin	−9.5
			Stigmasterol	−11.4

IL-6	1alu	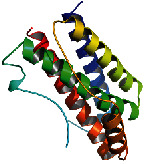	Quercetin	−7.2
			Luteolin	−7.2
			Kaempferol	−6.4
			Beta-sitosterol	−5.9
			Isorhamnetin	−6.5
			Stigmasterol	−6.4

MAPK1	6slg	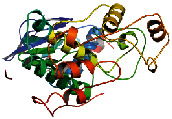	Quercetin	−7.6
			Luteolin	−8.3
			Kaempferol	−7.7
			Beta-sitosterol	−8.9
			Isorhamnetin	−8.4
			Stigmasterol	−8.9

TP53	6gga	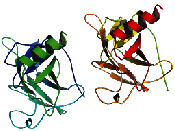	Quercetin	−7.5
			Luteolin	−8.1
			Kaempferol	−7.5
			Beta-sitosterol	−8.1
			Isorhamnetin	−7.5
			Stigmasterol	−8.1

DRD2	6cm4	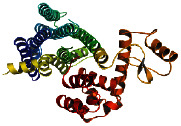	Quercetin	−7.4
			Luteolin	−7.4
			Kaempferol	−7.0
			Beta-sitosterol	−7.3
			Isorhamnetin	−6.9
			Stigmasterol	−7.4
NR3C1	6dxk	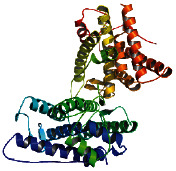	Quercetin	−8.6
			Luteolin	−8.5
			Kaempferol	−8.6
			Beta-sitosterol	−7.6
			Isorhamnetin	−8.7
			Stigmasterol	−8.4

**Table 4:** Binding free energy (kJ/mol) for 6hhi_G4N and 6hhi_Quercetin.

	van der Waals energy	Electrostatic energy	Polar solvation energy	SASA energy	Binding energy
6hhi_Quercetin	−165.732 ± 6.874	−9.592 ± 6.444	87.837 ± 8.989	−15.658 ± 0.811	−103.144 ± 10.692
6hhi_G4N	−343.293 ± 8.130	−74.817 ± 10.183	325.211 ± 11.934	−32.623 ± 0.832	−125.522 ± 14.620

## Data Availability

The datasets used and/or analyzed during the current study are available from the corresponding author on reasonable request.

## Abbreviations

DALY: Disability-adjusted life year
TCM: Traditional Chinese medicine
CCHP: Cyperi Rhizoma-Chuanxiong Rhizoma herb pair
PKA: Protein kinase A
CREB: cAMP response element-binding protein
NMDA: N-Methyl-D-aspartate
Akt: Protein kinase B
mTOR: Mammalian target of rapamycin
BDNF: Brain-derived neurotrophic factor
TCMSP: Traditional Chinese Medicine Systems Pharmacology Database and Analysis Platform
DL: Drug-likeness
OB: Oral bioavailability
STITCH: Search tool for interacting chemicals
TTD: Therapeutic target database
STRING: Search Tool for the Retrieval of Interacting Genes/Proteins
PPI: Protein-protein interaction
GO: Gene ontology
KEGG: Kyoto Encyclopedia of Genes and Genomes
DAVID: Database for Annotation, Visualization, and Integrated Discovery
FDR: False discovery rate
PDB: Protein Data Bank
AKT1: RAC-alpha serine/threonine-protein kinase
IL-6: Interleukin-6
TP53: Cellular tumor antigen p53
DRD2: D(2) dopamine receptor
MAPK1: Mitogen-activated protein kinase 1
NR3C1: Glucocorticoid receptor
TNF: Tumor necrosis factor
ESR1: Estrogen receptor
SST: Somatostatin
OPRM1: Mu-type opioid receptor
DRD3: D(3) dopamine receptor
ADRA2A: Alpha-2A adrenergic receptor
ADRA2C: Alpha-2C adrenergic receptor
IL-10: Interleukin-10
IL-1B: Interleukin-1 beta
IFN-G: Interferon-gamma
GSK3B: Glycogen synthase kinase-3 beta
PTEN: Phosphatidylinositol 3,4,5-trisphosphate 3-phosphatase and dual-specificity protein phosphatase PTEN
IGF1: Insulin-like growth factor I
HTR2A: 5-Hydroxytryptamine receptor 2A
MTOR: Serine/threonine-protein kinase mTOR
CHRM5: Muscarinic acetylcholine receptor M5
HTR2C: 5-Hydroxytryptamine receptor 2C
SLC6A3: Sodium-dependent dopamine transporter
CRP: C-Reactive protein
APOE: Apolipoprotein E
SOD1: Superoxide dismutase [Cu-Zn]
MAOA: Amine oxidase [flavin-containing] A
MAOB: Amine oxidase [flavin-containing] B
NOS1: Nitric oxide synthase, brain
NR3C2: Mineralocorticoid receptor
SLC6A4: Sodium-dependent serotonin transporter
CHRNA2: Neuronal acetylcholine receptor subunit alpha-2
COL1A1: Collagen alpha-1(I) chain
CYP2B6: Cytochrome P450 2B6
DRD1: D(1A) dopamine receptor
GABRA1: Gamma-aminobutyric acid receptor subunit alpha-1
GRIA2: Glutamate receptor 2
HTR3A: 5-Hydroxytryptamine receptor 3A
SLC6A2: Sodium-dependent noradrenaline transporter
HIF-1: Hypoxia-inducible factor-1
TrkB: Tropomyosin-related kinase B
Erk: Extracellular signal-regulated kinase
TNFR1: Tumor necrosis factor receptor 1
NF-*κ*B: Nuclear factor-*κ*B
BP: Biological process
CC: Cellular component
MF: Molecular function
PI3K: Phosphatidylinositol 3-kinase
MD: Molecular dynamics
LINCS: LINear Constraint Solver
PME: Particle mesh Ewald
NVT: Canonical ensemble
NPT: Constant pressure-constant temperature ensemble
VMD: Visual molecular dynamics
MMPBSA: Molecular mechanics Poisson–Boltzmann surface area
RMSD: Root-mean-square deviation
RMSFs: Root-mean-square fluctuations.

## Supplementary Materials

Supplementary Materials Additional File 1: Table S1: targets related to depression. Additional File 2: Fig. S1: Venn diagram of targets of CCHP and depression.
