# Supplementary material for: Network Pharmacology and Molecular Docking Analyses of Mechanisms Underlying Effects of the Cyperi Rhizoma-Chuanxiong Rhizoma Herb Pair on Depression
Source: Evid Based Complement Alternat Med. 2021 Dec 22;2021:5704578. doi: 10.1155/2021/5704578 (PMC8716227; doi:10.1155/2021/5704578)
Supplement: Supplementary Materials — Additional File 1: Table S1: targets related to depression. Additional File 2: Fig. S1: Venn diagram of targets of CCHP and depression. [file 5704578.f1.zip › 5704578.f1/Additional file 1 Table S1. Targets related to depression.pdf]

Table S1. Targets related to depression

Gene symbol	Protein name	Uniprot ID
BDNF	Brain-derived neurotrophic factor, BDNF	P23560
CRHR1	Corticotropin-releasing factor receptor 1	P34998
GABRA1	Gamma-aminobutyric acid receptor subunit alpha-1	P14867
GABRD	Gamma-aminobutyric acid receptor subunit delta	O14764
GRIA1	Glutamate receptor 1	P42261
GRIA2	Glutamate receptor 2	P42262
GRIA3	Glutamate receptor 3	P42263
GRM1	Metabotropic glutamate receptor 1	Q13255
HTR1A	5-hydroxytryptamine receptor 1A, 5-HT-1A	P08908
HTR2A	5-hydroxytryptamine receptor 2A	P28223
HTR2C	5-hydroxytryptamine receptor 2C, 5-HT-2C	P28335
MAOA	Amine oxidase [flavin-containing] A	P21397
MAOB	Amine oxidase [flavin-containing] B	P27338
NR3C1	Glucocorticoid receptor	P04150
ACE	Angiotensin-converting enzyme, ACE	P12821
ACTB	Actin, cytoplasmic 1	P60709
ADAM10	Disintegrin and metalloproteinase domain-containing protein 10	O14672
ADRA2A	Alpha-2A adrenergic receptor	P08913
ADRA2C	Alpha-2C adrenergic receptor	P18825
AKT1	RAC-alpha serine/threonine-protein kinase	P31749
ALB	Albumin	P02768
ALG9	Alpha-1,2-mannosyltransferase ALG9	Q9H6U8
APOE	Apolipoprotein E	P02649
ARF1	ADP-ribosylation factor 1	P84077
ARSA	ATPase GET3	O43681
ATP13A2	Polyamine-transporting ATPase 13A2	Q9NQ11
ATP1A3	Sodium/potassium-transporting ATPase subunit alpha-3	P13637
ATP2A2	Sarcoplasmic/endoplasmic reticulum calcium ATPase 2	P16615
AVP	Vasopressin-neurophysin 2-copeptin	P01185
BDKRB1	B1 bradykinin receptor	P46663
BRAF	Serine/threonine-protein kinase B-raf	P15056
C9orf72	Guanine nucleotide exchange C9orf72	Q96LT7
CACNA1A	Voltage-dependent P/Q-type calcium channel subunit alpha-1A	O00555
CACNA1C	Voltage-dependent L-type calcium channel subunit alpha-1C	Q13936
CBLN1	Cerebellin-1	P23435
CHAT	Choline O-acetyltransferase	P28329
CHMP2B	Charged multivesicular body protein 2b	Q9UQN3
CHRM5	Muscarinic acetylcholine receptor M5	P08912

CHRNA2	Neuronal acetylcholine receptor subunit alpha-2	Q15822
CLOCK	Circadian locomoter output cycles protein kaput	O15516
CNR1	Cannabinoid receptor 1	P21554
CNTNAP2	Contactin-associated protein-like 2	Q9UHC6
COL11A1	Collagen alpha-1(XI) chain	P12107
COL11A2	Collagen alpha-2(XI) chain	P13942
COL1A1	Collagen alpha-1(I) chain	P02452
COL2A1	Collagen alpha-1(II) chain	P02458
COMT	Catechol O-methyltransferase	P21964
CREB1	Cyclic AMP-responsive element-binding protein 1	P16220
CRH	Corticoliberin	P06850
CRP	C-reactive protein	P02741
CYP2B6	Cytochrome P450 2B6	P20813
CYP2D6	Cytochrome P450 2D6	P10635
DAO	D-amino-acid oxidase	P14920
DAOA	D-amino acid oxidase activator	P59103
DCTN1	Dynactin subunit 1	Q14203
DISC1	Disrupted in schizophrenia 1 protein	Q9NRI5
DLG3	Disks large homolog 3	Q92796
DNMT1	DNA (cytosine-5)-methyltransferase 1	P26358
DNMT3B	DNA (cytosine-5)-methyltransferase 3B	Q9UBC3
DPYSL2	Dihydropyrimidinase-related protein 2	Q16555
DRD1	D(1A) dopamine receptor	P21728
DRD2	D(2) dopamine receptor	P14416
DRD3	D(3) dopamine receptor	P35462
DRD4	D(4) dopamine receptor	P21917
EDNRA	Endothelin-1 receptor	P25101
EPO	Erythropoietin	P01588
EPOR	Erythropoietin receptor	P19235
ESR1	Estrogen receptor	P03372
FGF8	Fibroblast growth factor 8	P55075
FGFR1	Fibroblast growth factor receptor 1	P11362
FGFR2	Fibroblast growth factor receptor 2	P21802
FGFR3	Fibroblast growth factor receptor 3	P22607
FKBP5	Peptidyl-prolyl cis-trans isomerase FKBP5	Q13451
FLNA	Filamin-A	P21333
FMR1	Synaptic functional regulator FMR1	Q06787
GABBR1	Gamma-aminobutyric acid type B receptor subunit 1	Q9UBS5
GABBR2	Gamma-aminobutyric acid type B receptor subunit 2	O75899
GABRB2	Gamma-aminobutyric acid receptor subunit beta-2	P47870
GABRG2	Gamma-aminobutyric acid receptor subunit gamma-2	P18507
GABRG3	Gamma-aminobutyric acid receptor subunit gamma-3	Q99928
GAD1	Glutamate decarboxylase 1	Q99259
GBA	Lysosomal acid glucosylceramidase	P04062

GCH1	GTP cyclohydrolase 1	P30793
GDNF	Glial cell line-derived neurotrophic factor	P39905
GFAP	Glial fibrillary acidic protein	P14136
GHRL	Appetite-regulating hormone	Q9UBU3
GLI2	Zinc finger protein GLI2	P10070
GLI3	Transcriptional activator GLI3	P10071
GNAS	Guanine nucleotide-binding protein G(s) subunit alpha isoforms XLas	Q5JWF2
GNB3	Guanine nucleotide-binding protein G(I)/G(S)/G(T) subunit beta-3	P16520
GNRH1	Progonadoliberin-1	P01148
GRIA4	Glutamate receptor 4	P48058
GRID2	Glutamate receptor ionotropic, delta-2	O43424
GRIK2	Glutamate receptor ionotropic, kainate 2	Q13002
GRIN1	Glutamate receptor ionotropic, NMDA 1	Q05586
GRIN2A	Glutamate receptor ionotropic, NMDA 2A	Q12879
GRIN2B	Glutamate receptor ionotropic, NMDA 2B	Q13224
GRM2	Metabotropic glutamate receptor 2	Q14416
GRM5	Metabotropic glutamate receptor 5	P41594
GRM7	Metabotropic glutamate receptor 7	Q14831
GRN	Progranulin	P28799
GSK3B	Glycogen synthase kinase-3 beta	P49841
H1R	Histamine H1 receptor	P35367
HCRT	Orexin	O43612
HLA-DQB1	HLA class II histocompatibility antigen, DQ beta 1 chain	P01920
HLA-DRB1	HLA class II histocompatibility antigen, DRB1 beta chain	P01911
HRAS	GTPase HRas	P01112
HTR1B	5-hydroxytryptamine receptor 1B	P28222
HTR2B	5-hydroxytryptamine receptor 2B	P41595
HTR3A	5-hydroxytryptamine receptor 3A	P46098
HTT	Huntingtin	P42858
IDO1	Indoleamine 2,3-dioxygenase 1	P14902
IDUA	Alpha-L-iduronidase	P35475
IFNG	Interferon gamma	P01579
IGF1	Insulin-like growth factor I	P05019
IL10	Interleukin-10	P22301
IL1B	Interleukin-1 beta	P01584
IL6	Interleukin-6	P05231
INS	Insulin	P01308
ITIH3	Inter-alpha-trypsin inhibitor heavy chain H3	Q06033
KCNQ3	Potassium voltage-gated channel subfamily KQT member 3	O43525
KDM6A	Lysine-specific demethylase 6A	O15550
KRAS	GTPase KRas	P01116

LEP	Leptin	P41159
LRRK2	Leucine-rich repeat serine/threonine-protein kinase 2	Q5S007
MAP2K1	Dual specificity mitogen-activated protein kinase kinase 1	Q02750
MAP2K2	Dual specificity mitogen-activated protein kinase kinase 2	P36507
MAPK1	Mitogen-activated protein kinase 1	P28482
MAPT	Microtubule-associated protein tau	P10636
MCHR1	Melanin-concentrating hormone receptor 1	Q99705
MECP2	Methyl-CpG-binding protein 2	P51608
MOP	Melanopsin	Q9UHM6
MTHFR	Methylenetetrahydrofolate reductase	P42898
MTOR	Serine/threonine-protein kinase mTOR	P42345
NaC	NACHT	Q9C000
NET	Ephrin type-B receptor 1	P54762
NGF	Beta-nerve growth factor	P01138
NOS1	Nitric oxide synthase, brain	P29475
NOTCH3	Neurogenic locus notch homolog protein 3	Q9UM47
NPAS2	Neuronal PAS domain-containing protein 2	Q99743
NPY	Pro-neuropeptide Y	P01303
NR1D1	Nuclear receptor subfamily 1 group D member 1	P20393
NR3C2	Mineralocorticoid receptor	P08235
NR4A2	Nuclear receptor subfamily 4 group A member 2	P43354
NRXN1	Neurexin-1	Q9ULB1
NTRK2	BDNF/NT-3 growth factors receptor	Q16620
OPRL1	Nociceptin receptor	P41146
OPRM1	Mu-type opioid receptor	P35372
OXT	Oxytocin-neurophysin 1	P01178
OXTR	Oxytocin receptor	P30559
PAH	Phenylalanine-4-hydroxylase	P00439
PANK2	Pantothenate kinase 2	Q9BZ23
PARK7	Parkinson disease protein 7	Q99497
PCLO	Protein piccolo	Q9Y6V0
PER2	Period circadian protein homolog 2	O15055
PER3	Period circadian protein homolog 3	P56645
PICK1	PRKCA-binding protein	Q9NRD5
PLA2G6	85/88 kDa calcium-independent phospholipase A2	O60733
PLK2	Serine/threonine-protein kinase PLK2	Q9NYY3
POLG	DNA polymerase subunit gamma-1	P54098
POMC	Pro-opiomelanocortin	P01189
POU1F1	Pituitary-specific positive transcription factor 1	P28069
PRKAR1A	cAMP-dependent protein kinase type I-alpha regulatory subunit	P10644
PRKCG	Protein kinase C gamma type	P05129
PRKN	E3 ubiquitin-protein ligase parkin	O60260
PRL	Prolactin	P01236

PRNP	Major prion protein	P04156
PSEN1	Presenilin-1	P49768
PTCH1	Protein patched homolog 1	Q13635
PTEN	Phosphatidylinositol 3,4,5-trisphosphate 3-phosphatase and dual-specificity protein phosphatase PTEN	P60484
PTH	Parathyroid hormone	P01270
RELN	Reelin	P78509
SCN11A	Sodium channel protein type 11 subunit alpha	Q9UI33
SGCE	Epsilon-sarcoglycan	O43556
SHANK3	SH3 and multiple ankyrin repeat domains protein 3	Q9BYB0
SHH	Sonic hedgehog protein	Q15465
SLC18A2	Synaptic vesicular amine transporter	Q05940
SLC6A2	Sodium-dependent noradrenaline transporter	P23975
SLC6A3	Sodium-dependent dopamine transporter	Q01959
SLC6A4	Sodium-dependent serotonin transporter	P31645
SMPD4	Sphingomyelin phosphodiesterase 4	Q9NXE4
SNCA	Alpha-synuclein	P37840
SOD1	Superoxide dismutase [Cu-Zn]	P00441
SST	Somatostatin	P61278
SYN2	Synapsin-2	Q92777
SYP	Synaptophysin	P08247
TACR1	Substance-P receptor, SPR	P25103
TACR2	Substance-K receptor, SKR	P21452
TACR3	Neuromedin-K receptor, NKR	P29371
TARDBP	TAR DNA-binding protein 43	Q13148
TH	Tyrosine 3-monooxygenase	P07101
TMEM106B	Transmembrane protein 106B	Q9NUM4
TNF	Tumor necrosis factor	P01375
TOR1A	Torsin-1A	O14656
TP53	Cellular tumor antigen p53	P04637
TPH1	Tryptophan 5-hydroxylase 1	P17752
TPH2	Tryptophan 5-hydroxylase 2	Q8IWU9
TRPC4	Short transient receptor potential channel 4	Q9UBN4
TRPC5	Short transient receptor potential channel 5	Q9UL62
TRPV1	Transient receptor potential cation channel subfamily V member 1	Q8NER1
TWIST2	Twist-related protein 2	Q8WVJ9
TWNK	Twinkle protein, mitochondrial	Q96RR1
TYMP	Thymidine phosphorylase	P19971
VCP	Transitional endoplasmic reticulum ATPase	P55072
WASHC5	WASH complex subunit 5	Q12768
WFS1	Wolframin	O76024
XBP1	X-box-binding protein 1	P17861
