# Supplementary figures and images for: Network Pharmacology and Molecular Docking Analyses of Mechanisms Underlying Effects of the Cyperi Rhizoma-Chuanxiong Rhizoma Herb Pair on Depression
Source: Evid Based Complement Alternat Med. 2021 Dec 22;2021:5704578. doi: 10.1155/2021/5704578 (PMC8716227; doi:10.1155/2021/5704578)

CCHP

depression

275  
(57.1%)

40  
(8.3%)

167  
(34.6%)

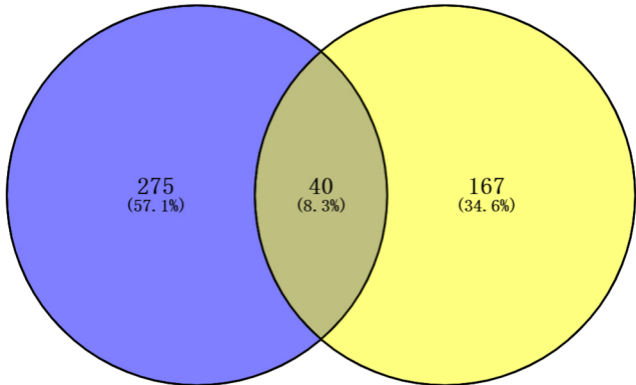

Supplement: Supplementary Materials — Additional File 1: Table S1: targets related to depression. Additional File 2: Fig. S1: Venn diagram of targets of CCHP and depression. [file 5704578.f1.zip › 5704578.f1/Additional file 2 Fig. S1. Venn diagram of targets of CCHP and depression.pdf]
